# Nutrient availability affects the polar lipidome of *Halimione portulacoides* leaves cultured in hydroponics

**DOI:** 10.1038/s41598-020-63551-1

**Published:** 2020-04-20

**Authors:** Marco Custódio, Elisabete Maciel, Maria Rosário Domingues, Ana Isabel Lillebø, Ricardo Calado

**Affiliations:** 1 ECOMARE, Centre for Environmental and Marine Studies (CESAM), Department of Biology, University of Aveiro, Santiago University Campus, 3810-193 Aveiro, Portugal; 2 ECOMARE, Centre for Environmental and Marine Studies (CESAM), Department of Chemistry, University of Aveiro, Santiago University Campus, 3810-193 Aveiro, Portugal; 3 Mass Spectrometry Center, Department of Chemistry & QOPNA & LAQV - Requinte, University of Aveiro, Campus Universitário de Santiago, 3810-193 Aveiro, Portugal

**Keywords:** Lipidomics, Lipids, Mass spectrometry, Plant stress responses

## Abstract

Halophytes are increasingly regarded as suitable extractive species and co-products for coastal Integrated Multi-Trophic Aquaculture (IMTA) and studying their lipidome is a valid means towards their economic valorization. *Halimione portulacoides* (L.) Aellen edible leaves are rich in functional lipids with nutraceutical and pharmaceutical relevance and the present study aimed to investigate the extent to which its lipidome remains unchanged under a range of dissolved inorganic nitrogen (N) and phosphorus (P) concentrations typical of aquaculture effluents. Lipidomics analysis, done by hydrophilic interaction liquid chromatography coupled to high resolution mass spectrometry, identified 175 lipid species in the lipid extract of leaves: 140 phospholipids (PLs) and 35 glycolipids (GLs). Plants irrigated with a saline solution with 20–100 mg DIN-N L^−1^ and 3–15.5 mg DIP-P L^−1^ under a 1-week hydraulic retention time displayed a relatively stable lipidome. At lower concentrations (6 mg DIN-N L^−1^ and 0.8 mg DIP-P L^−1^), plants exhibited less PLs and GLs per unit of leaves dry weight and the GLs fraction of the lipidome changed significantly. This study reveals the importance of analyzing the lipidomic profile of halophytes under different nutritional regimens in order to establish nutrient-limitation thresholds and assure production conditions that deliver a final product with a consistent lipid profile.

## Introduction

Halophyte plants display unique physiological and ecological adaptations to salt-marsh ecosystems, which allow them to live and thrive under a wide range of salt concentrations that most plants are unable to tolerate^1–4^. These plants have been investigated in several contexts, providing important insights on salt-tolerance mechanisms in order to improve salt-sensitive crops^5–7^. Moreover, their potential as alternative agricultural crops have also been investigated for multiple applications^8–12^. In the context of aquaculture, recent studies have been testing the integration of halophytes production as an approach to extract nutrients from nutrient-rich saline effluents produced by fish-farming activities, which have been recently reviewed by Custódio *et al*. (2017)^13^. These investigations are typically performed in the context of Integrated Multi-Trophic Aquaculture (IMTA), a conceptual production model regarded as a more sustainable solution for the aquaculture industry^14–17^.

Entrepreneurs and society in general are only recently realizing the potential of halophytes as crops for the future and, besides the more obvious suitability of a handful of species for direct human and animal consumption (e.g. fresh/dried produce, plant meal), a particularly interesting market-positioning strategy for added-value could be the pharmaceutical and nutraceutical industries. Recent studies demonstrated that the leaves from certain halophyte are rich in bioactive molecules, such as phenols, flavonoids and other lipophilic compounds^8,18–23^.

Marine lipids are regarded as an untapped pool of molecules with nutraceutical and pharmaceutical potential, especially those from marine macrophytes^10,22,24–27^. Glycolipids (GLs) and phospholipids (PLs) present in seaweeds (e.g. *Codium tomentosum* Stackhouse*, Gracilaria* spp., *Porphyra dioica* J. Brodie & L. M. Irvine) displayed antioxidant, anti-inflammatory and antibacterial properties and their fatty acid composition is rich in polyunsaturated aliphatic chains, which increase their functional properties for human health^20,21,28–30^. Several bioactive properties have been related to GLs and PLs (e.g. anti-inflammatory and anticarcinogenic) as well as enhanced human cognitive functions and motor performance^28,31–36^. Halophytes, contrarily to algae, have been particularly overlooked on that regard, and the existing lipid characterizations have been mostly limited to fatty acids, non-polar lipids and sterols^19,22,37,38^. To date, only one publication attempted to describe the polar lipidome of two edible halophyte species (*Salicornia ramosissima* J. Woods and *Halimione portulacoides* (L.) Aellen), using liquid-chromatography coupled with mass-spectrometry (LC-MS)^22^. Fully exploring the lipidome of halophytes is a major step towards their valorization as relevant cash-crops for both agriculture and aquaculture. Besides it is also important to take into consideration the potential variations in their lipidomic profile in response to changes in environmental and metabolic conditions^10,37,39–41^. Understanding the circumstances and extent of those variations is essential to guarantee the supply of a consistent product when a stable lipid profile is a requisite.

The present study aimed to describe and assess potential shifts in the lipidome of sea purslane *H. portulacoides* leaves, grown hydroponically under different concentrations of dissolved inorganic nitrogen (DIN) and phosphorous (DIP). The concentrations used in this study aim to represent a wide range of possible values, as recorded in aquaculture effluents used in previous halophyte bioremediation studies under IMTA conditions^42–46^. To understand if contrasting concentrations of DIN and DIP affect the polar lipidome of *H. portulacoides* leaves, the present study tested the following null hypothesis (H_0_): ‘There are no significant changes in the polar lipidome of *H. portulacoides* cultivated in low-, medium- and high-input of DIN and DIP’. Lipid profile was evaluated by state of the art lipidomics analysis using HILIC coupled with mass spectrometry (MS) and tandem mass spectrometry (MS/MS), bioinformatic tools and statistical analysis.

## Results

### Total lipids, glycolipids and phospholipids quantification

**Figure 1:**
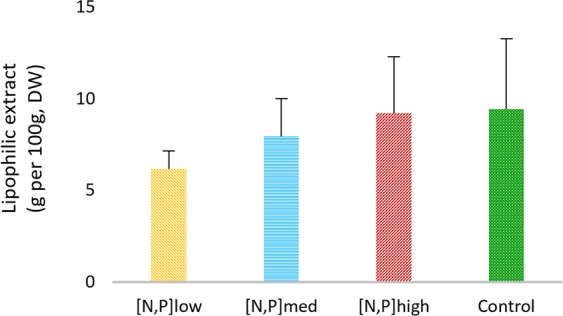
Total amount of the lipid extract of *Halimione portulacoides* leaves. Error bars represent standard deviations.

Total lipid content was estimated by gravimetry and expressed as *g* 1*00 g*^*−*1^ of dry weight (DW) (Fig. 1). Non-significant differences were detected between each treatment and the control (CT), with a tendency for increased lipid content in the leaves of *H. portulacoides* at higher concentrations of N and P in the solution: [N,P]_low_ yielded 6,18 ± 0,99 g 100 g^−1^ of leaves dry weight (DW), followed by [N,P]_med_, with 7.96 ± 2.05 g 100 g^−1^ DW; and 9.23 ± 3.06 g 100 g^−1^ DW for [N,P]_high_. The total amount of lipid extract obtained from [N,P]_high_ was similar to that recorded in the CT (9.44 ± 3.83 g 100 g^−1^ DW).

**Figure 2:**
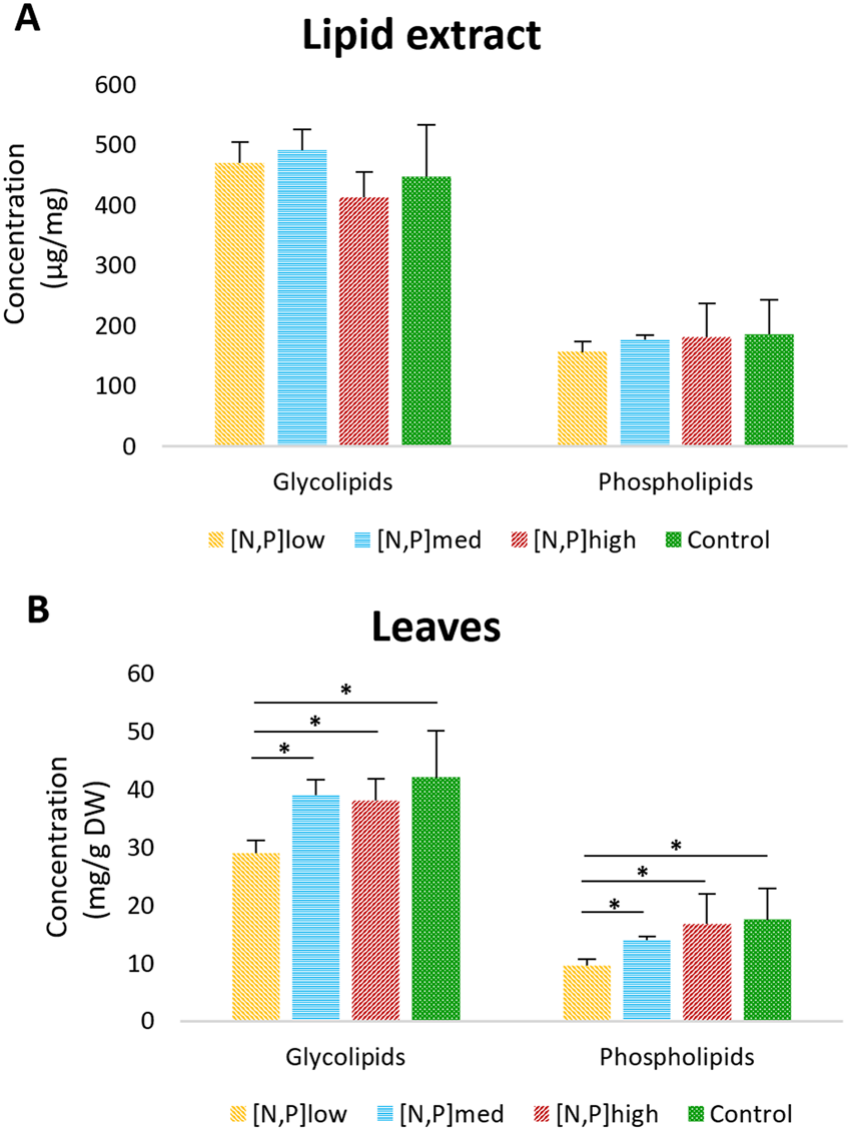
Glycolipids and phospholipids concentrations in (**A**) leaves lipid extract and in (**B**) leaves dry mass of *Halimione portulacoides*. Error bars represent standard deviations and horizontal lines with symbol * represent significant differences: *p < 0,05; **p < 0,01.

The levels of GLs and PLs present in the lipid extracts of the leaves of *H. portulacoides* were also estimated (Fig. 2A), expressed as *µg mg*^*−1*^ of lipid extract. No significant differences were detected between treatments in neither GLs nor PLs contents. The overall average content of GLs was 455.87 ± 57.32 µg mg^−1^ of lipid extract and that of PLs was 175.49 ± 39.56 µg mg^−1^ of lipid extract.

Significant differences were recorded between treatment conditions regarding the concentration of GLs and PLs in the leaves of *H. portulacoides*, expressed as *mg g*^*−1*^ DW (Fig. 2B). The [N, P]_low_ group had a significantly lower concentration of GLs (29.1 mg g^−1^ DW) than the CT (42.1 mg g^−1^ DW) and other treatments (38.2–39.1 mg g^−1^ DW), as well as a significantly lower concentration of PLs (9.7 mg g^−1^ DW) than the CT (17.5 mg g^−1^ DW) and other treatments (14.1–16.8 mg g^−1^ DW). The [N, P]_med_ and [N, P]_high_ groups did not differ from the CT in either type of lipids.

### Lipidomic signature

A non-targeted lipidomics approach was used to evaluate the stability of the lipidome across treatment conditions. This approach provided a global profile of the polar lipid molecular species present in the extracts and potentially used as a lipid signature that characterizes states of N and P limitation and/or excess.

**Figure 3:**
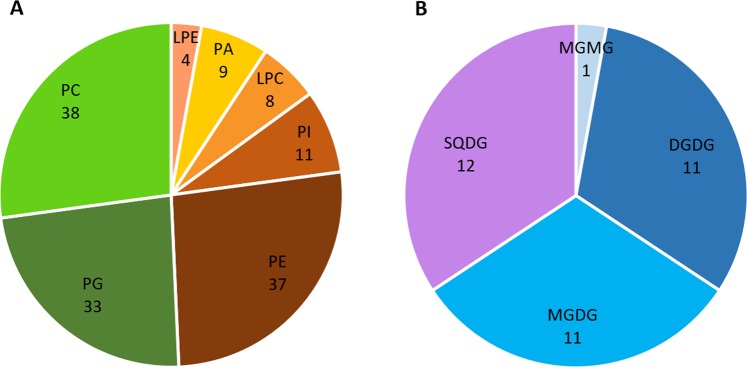
Number of (**A**) phospholipids and (**B**) glycolipids molecular species identified in the lipid extract of *Halimione portulacoides* leaves by MS/MS. DGDG – digalactosyldiacylglycerol; LPC – lysophosphatidylcholine; LPE – lysophosphatidylethanolamine; MGDG – monogalactosyldiacylglycerol; MGMG – monogalactosylmonoacylglycerol; PA – phosphatidic acid; PC – phosphatidylcholine; PG – phosphatidylglycerol; PE – phosphatidylethanolamine; PI – phosphatidylinositol; SQDG - sulfoquinovosyldiacylglycerol.

MS and MS/MS analysis allowed the accurate identification of 175 lipid species, namely 140 PLs (Table 1) and 35 GLs (Table 2), which were detected in all conditions. In a few cases, MS/MS spectra did not provide enough information to determine the fatty acyl composition, but the class was confirmed through the identification of the polar head and are therefore included in Table 1. No lipids were found to be unique to any one condition. The lipid classes identified were previously recorded in wild specimens^27^ and are DGDGs, LPCs, LPEs, MGDGs, MGMG, PAs, PCs, PEs, PGs, PIs and SQDGs. The number of species identified per lipid class is represented in Fig. 3.

**Table 1:** Phospholipids molecular species identified by LC-MS and tandem MS (MS/MS) from total lipid extracts of *Halimione portulacoides* leaves.

**[M + H]**^**+**^	**Lysophosphatidylcholine**	844.6785	PC 18:1/22:0
496.3396	LPC 16:0	868.6785	PC 18:3/24:0; PC 18:2/24:1
518.3230	LPC 18:3	870.6933	PC 18:2/24:0; PC 18:1/24:1
520.3387	LPC 18:2		
522.3564	LPC 18:1	**[M-H]**^**−**^	**Phosphatidylethanolamine**
550.3869	LPC 20:1	684.4609	PE 16:0/16:3; PE 14:0/18:3
552.4024	LPC 20:0	686.4753	PE 16:0/16:2; PE 14:0/18:2
580.4350	LPC 22:0	688.4914	PE 16:0/16:1; PE 14:0/18:1
608.4656	LPC 24:0	708.4596	PE 16:2/18:3; PE 16:3/18:2
		710.4754	PE 16:1/18:3; PE 16:2/18:2
**[M-H]**^**−**^	**Lysohosphatidylethanolamine**	712.4916	PE 16:0/18:3; PE 16:1/18:2
452.2779	LPE 16:0	714.5068	PE 16:0/18:2; PE 16:1/18:1
474.2621	LPE 18:3	716.522	PE 16:0/18:1
476.2779	LPE 18:2	734.4766	PE 18:3/18:3
478.2938	LPE 18:1	736.4921	PE 18:2/18:3
		738.5086	PE 18:2/18:2; PE 18:1/18:3
**[M-H]**^**−**^	**Phosphatidic acid**	740.5227	PE 18:1/18:2
667.4347	PA 34:4*	742.5387	PE 18:1/18:1
669.4505	PA 16:0/18:3	764.5219	PE 18:0/20:5
671.4647	PA 16:0/18:2	766.5389	PE 18:3/20:1; PE 18:2/20:2
673.4816	PA 16:0/18:1	768.5536	PE 18:2/20:1; PE 18:3/20:0; PE 18:1/20:2
691.4335	PA 18:3/18:3	770.5684	PE 18:1/20:1; PE 18:2/20:0
693.4498	PA 18:2/18:3	794.5699	PE 18:/22:1
695.4647	PA 18:2/18:2; PA 18:1/18:3	796.5857	PE 18:3/22:0; PE 18:2/22:1
697.4812	PA 18:1/18:2	798.5998	PE 18:2/22:0
699.4955	PA 18:1/18:1	800.6154	PE 18:1/22:0
		824.6159	PE 18:2/24:1; PE 18:3/24:0
**[M** + **H]**^**+**^	**Phosphatidylcholine**	826.6316	PE 18:2/24:0
700.4889	PC 30:3*		
728.5230	PC 16:0/16:3	**[M-H]**^**−**^	**Phosphatidylglycerol**
730.5378	PC 16:0/16:2; PC 14:0/18:2	693.4703	PG 14:0/16:0
734.5684	PC 16:0/16:0	719.486	PG 16:0/16:1; PG 14:0/18:1
750.5072	PC 18:3/18:3	721.5013	PG 16:0/16:0; PG 14:0/18:0
754.5373	PC 16:1/18:3; PC 16:2/18:2; PC 16:3/18:1	739.4554	PG 16:1/18:4; PG 16:2/18:3; PG 16:3/18:2
756.5538	PC 16:0/18:3; PC 16:1/18:2	741.4701	PG 16:0/18:4; PG 16:1/18:3; PG 16:2/18:2; PG 16:3/18:1
758.5690	PC 16:0/18:2; PC 16:1/18:1	743.4861	PG 16:0/18:3; PG 16:1/18:2; PG 16:2/18:1
760.5829	PC 16:0/18:1	745.5014	PG 16:1/18:1; PG 16:2/18:0; PG 16:0/18:2
772.4896	PC 36:9*	747.5162	PG 16:0/18:1; PG 16:1/18:0
776.5193	PC 36:7*	763.4543	PG 18:3/18:4
778.5370	PC 18:3/18:3	765.4716	PG 18:3/18:3; PG 18:2/18:4
780.5529	PC 18:2/18:3	767.4850	PG 18:2/18:3
782.5682	PC 18:2/18:2; PC 18:1/18:3	769.5012	PG 18:2/18:2; PG 18:1/18:3; PG 16:1/20:3
784.5841	PC 18:1/18:2; PC 18:0/18:3	771.5160	PG 18:1/18:2
786.5997	PC 18:1/18:1; PC 18:0/18:2	773.5316	PG 18:1/18:1; PG 18:0/18:2; PG 16:0/20:2
800.5198	PC 38:9*	775.5458	PG 16:0/20:1; PG18:0/18:1
802.5347	PC 38:8*		
804.5510	PC 38.7*	**[M-H]**^**−**^	**Phosphatidylinositol**
806.5667	PC 38:6*	831.5016	PI 16:0/18:3
808.5818	PC 18:2/20:3; PC 18:3/20:2	833.5165	PI 16:0/18:2
810.5978	PC 18:3/20:1; PC 18:2/20:2	835.5318	PI 16:0/18:1
812.6158	PC 18:2/20:1	853.4849	PI 18:3/18:3
814.6334	PC 18:1/20:1; PC 18:2/20:0	855.5002	PI 18:2/18:3
832.5815	PC 40:7*	857.5163	PI 18:2/18:2; PI 18:1/18:3
834.5967	PC 40:6*	859.5319	PI 18:1/18:2
838.6321	PC 18:3/22:1	861.5471	PI 18:1/18:1; PI 18:0/18:2
840.6473	PC 18:3/22:0; PC 18:2/22:1	863.5607	PI 18:1/18:0
842.6634	PC 18:2/22:0; PC 18:1/22:1		

*confirmed *m/z* and class but missing fatty-acyl information to identify species.

**Table 2:** Glycolipids molecular species identified by LC-MS and tandem MS (MS/MS) from total lipid extracts of *Halimione portulacoides* leaves.

[M + NH_4_]^+^	Digalactosyldiacylglycerol	[M + NH_4_]^+^	Monogalactosylmonoacylglycerol
910.6472	DGDG 16:0/16:0	532.3482	MGMG 18:3
926.5816	DGDG 18:3/16:3		
932.6296	DGDG 18:3/16:0; DGDG 18:2/16:1	**[M-H]**^**−**^	**Sulfoquinovosyldiacylglycerol**
936.6576	DGDG 18:1/16:0	787.4660	SQDG 18:3/14:0; SQDG 16:3/16:0
954.6144	DGDG 18:3/18:3; DGDG 18:4/18:2	789.4800	SQDG 18:2/14:0
958.6440	DGDG 18:3/18:1; DGDG 18:2/18:2	791.4970	SQDG 16:1/16:0
960.6601	DGDG 18:3/18:0; DGDG 18:2/18:1	793.5120	SQDG 16:0/16:0
		813.4820	SQDG 18:3/16:1
**[M** + **NH**_**4**_**]**^**+**^	**Monogalactosyldiacylglycerol**	815.4970	SQDG 18:3/16:0
764.5308	MGDG 18:3/16:3; MGDG 18:4/16:2; MGDG 18:2/16:4	837.4800	SQDG 18:3/18:3
768.5630	MGDG 18:3/16:1	839.4970	SQDG 18:3/18:2; SQDG 20:2/16:3
770.5768	MGDG 18:3/16:0; MGDG 18:2/16:1; MGDG 18:0/16:3	843.5280	SQDG 18:3/18:0; SQDG 18:2/18:1
792.5615	MGDG 18:3/18:3; MGDG 18:4/18:2		
796.5910	MGDG 18:2/18:2; MGDG 18:3/18:1

**Figure 4:**
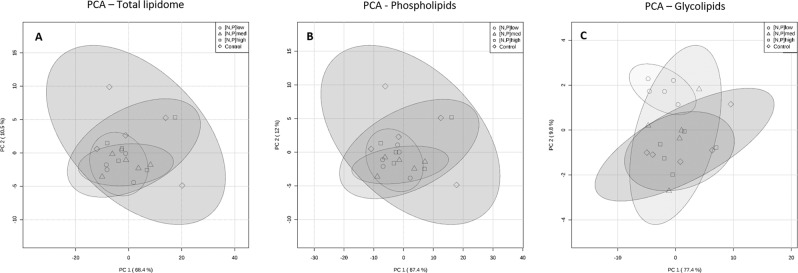
Principal component analysis **(**PCA) scores plot of (**A**) total lipidome, (**B**) phospholipids and (**C**) glycolipids normalized peak-intensity, obtained from the lipid extracts from *Halimione portulacoides* leaves.

After raw data processing and species identification, the dataset was analyzed using chemometric statistical methods to extract and interpret data from a biologically relevant perspective, looking at changes in the lipidome in general and within specific lipid groups (GLs and PLs). A PCA analysis was applied to a matrix with all lipid species, to highlight possible changes in the total lipidome imposed by treatments, from which scores plot (Fig. 4A) and loadings plot (supplementary Figure S1-A available as Supplementary Material) of the two principal components were obtained. PCA did not differentiate treatment conditions and there was a higher degree of variability within the CT group (and, to a lesser extent, in [N,P]_high_) compared with the other treatments.

**Figure 5:**
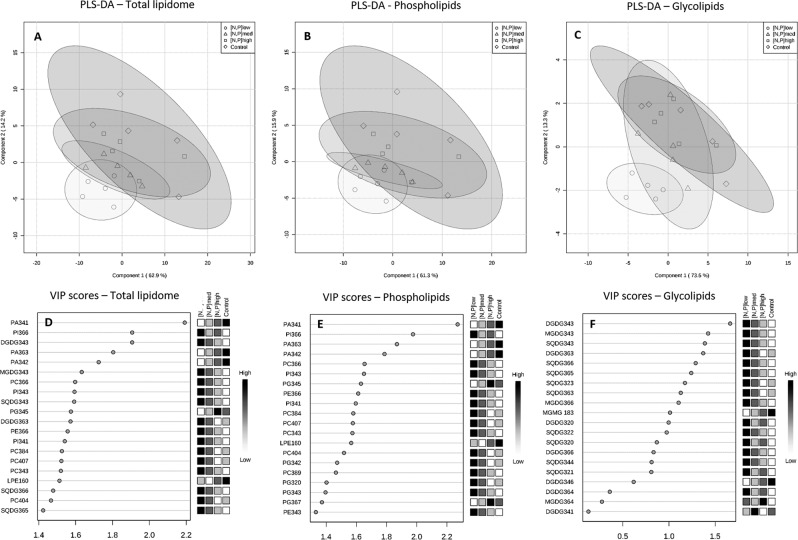
Partial least squares – discriminant analysis **(**PLS-DA) plots of (**A**) total lipidome, (**B**) phospholipids and (**C**) glycolipids peak-intensity matrices detected in the lipid extract from *Halimione portulacoides* leaves. The Variable Importance in the Projection (VIP) scores of each PLS-DA model are displayed below each plot (top 20 variables): (**D**) total lipidome, (**E**) phospholipids and (**F**) glycolipids.

Following this observation, PLS-DA was used to maximize the separation between conditions and the projection plot (Fig. 5A) revealed some degree of discrimination between [N,P]_low_ and both CT and [N,P]_high_. There was no discrimination between CT and both [N,P]_med_ and [N,P]_high_. The Variable Importance in Projection (VIP) scores were used to rank variables in terms of their importance in the projection of the PLS model, and the top 20 variables are presented in Fig. 5D. Fifteen out of those twenty species presented higher concentrations in [N,P]_low_ than CT and [N,P]_high_. Nonetheless, within the top five species explaining the separation, three were at lowest concentrations (PA 34:1, PA 36:3, PA 34:2) and two at highest concentrations (PI 36:6, DGDG 34:3) in [N,P]_low._

PCA and PLS-DA were also applied to matrices composed of only PL or GL molecular species, in order to decipher if any one of these two major lipid groups were changing more markedly than the other. The PCA of PLs (Fig. 4B; loadings in supplementary Figure S1-B) showed a very similar trend to the PCA plotted for the total lipidome data, suggesting no clear discrimination between treatment conditions. Similarly, the PLS-DA (Fig. 5B) also resembles the one obtained with the total lipidome matrix, with the most important species influencing the model also being PA 34:1, PA 36:3, PA 34:2 and PI 36:6 (Fig. 5E).

The PCA of GLs (Fig. 4C; loadings in supplementary Figure S1-C), on the other hand, evidences a clear separation between the [N,P]_low_ group and both CT and [N,P]_high_ groups. The PLS-DA model projection (Fig. 5C) further discriminates those groups, meanwhile the [N,P]_med_ group intersects both low-input and high-input clusters. The species that most contributes to the separation is DGDG 34:3, followed by MGDG 34:3, SQDG 34:3 and DGDG 36:3, all of them at higher concentrations in [N,P]_low_ than CT and [N,P]_high_ (Fig. 5F). From the top fifteen GL species influencing the PLS-DA model, fourteen were at highest concentration in [N,P]_low_.

The univariate analysis of individual lipid species intensities (supplementary Figure S2) displayed significant differences between certain treatment groups for PA 34:1, DGDG 34:3, DGDG 36:3 and PI 36:6, all of which were at the top of the VIP scores. MS/MS spectra from all conditions for some of the top VIP features can be consulted in the supplementary Figure S3.

### Polar lipid changes in each class

A univariate analysis was also performed regarding the average relative abundance of molecular species within each class of PLs and GLs. In the case of PLs, most of the significant differences in species relative abundance were observed between [N,P]_low_ and either CT or [N,P]_high_ or both conditions. These were observed in PCs (8 species), PEs (2), PGs (8), PIs (5), LPCs (2) and LPEs (2) (supplementary Figure S4). In PAs, 5 species were significantly different in terms of relative abundance between CT and the other treatments. In the case of GLs, differences were mostly observed between [N,P]_low_ and either CT or [N,P]_high_ or both, in DGDGs (4 species), MGDGs (4) and SQDGs (5) (supplementary Figure S5).

Some lipid species with similar fatty acyl chains were more abundant in [N,P]_low_, such as PC, PE, PG, PI, DGDG and SQDG with 34 carbons and 3 double bonds (34:3) and the PC, PE, PA and DGDG with 36 carbons and 6 double bonds (36:6). Regarding lyso-forms, the LPC 18:3 and LPE 18:3 were also more abundant in [N,P]_low_. The lipid species that were lower in [N,P]_low_ when compared with CT were more diversified in their fatty acyl composition and included several PLs (e.g. PC and PA 36:3, PC 32:2, PA 34:2, PI and PG 32:1), two lyso-PLs (LPC and LPE 16:0) and some GLs (e.g. MGDG 34:6, DGDG 34:6 and, SQDG 34:4). Other differences were observed between the [N,P]_low_ and [N,P]_high_ conditions: [N,P]_low_ treatment resulted in significantly lower relative abundance of PG 32:1 and 34:4, PI 34:2, SQDG 32:0, 32:1, 32:2 and 34:4, MGDG 34:3, 34:4, 36:4, DGDG 34:6 and 36:6; and higher relative abundance of PG 34:2, PI 34:1, SQDG 34:3, DGDG 34:3, 34:6 and 36:3.

## Discussion

The present study evaluated the polar lipidome signature of leaves from hydroponically grown *H. portulacoides* under different concentrations of N and P. The aim was to describe and reveal possible shifts in the lipidomic profile of its leaves under a wide range of DIN and DIP concentrations that represent possible IMTA contexts.

The total lipids extracted from *H. portulacoides* leaves ranged, on average, between 6.2–9.4 g 100 g^−1^ DW, with higher amounts extracted from the high-input treatments, including CT (Hoagland’s solution). A recent study by Patel *et al*. (2019)^47^ analyzed the total lipid content in the shoots (which can include leaves and other superior organs) of several halophytes and concluded that non-succulent halophytes (e.g. *Sporobolus virginicus* (L.) Kunth and *Aeluropus lagopoides* (L.) Trin. ex Thwaites) presented higher lipid content, between 5.5–7.2 g 100 g^−1^fresh weight (FW), followed by shrubby halophytes (e.g. *Atriplex nummularia* Lindl. and *Atriplex griffithii* Moq.) with 2.6–2.8 g 100 g^−1^ FW, and succulents (e.g. *Sesuvium portulacastrum* (L.) L. and *Salicornia brachiate* Miq.), with 1.5–1.8 g 100 g^−1^ FW. Given *H. portulacoides* leaves have a moisture content of ~90%, the equivalent amount of lipid extract in fresh leaves ranges from 0.76–0.94 g 100 g^−1^ FW, which is much lower than the values mentioned above for other species. Nonetheless, other studies reported values (both in DW and FW) within the same order of magnitude as *H. portulacoides*, which contradict the values reported above. For instance, *Salicornia bigelovii* Torr. was reported to have 0.37 g of total lipids 100 g^−1^ FW^48^; *S. ramosissima*, 1.87 g 100 g^−1^ DW^11^; *Sarcocornia perennis* (Mill.) A. J. Scott, 2.25 g 100 g^−1^ DW^11^ and *Crithmum maritimum* L., 1.53–2.16 g 100 g^−1^ DW^49^. This discrepancy could be explained by the inclusion of seeds along with the shoots in Patel *et al*. (2019), which would substantially increase total lipid content. Yet, total lipids in fertile shoot segments containing seeds were described in *Sarcocornia ambigua* (Michx.) M.A. Alonso & M.B. Crespo at concentrations between 1.4–5.2 g 100 g^−1^ DW^50^, in *Salicornia virginica* L. 2.4–3.6 g 100 g^−1^ DW^51^ and in *Salicornia europaea* L. 3.5–7.1 g 100 g^−1^ DW^51^, values that do not match up with the aforementioned concentrations reported for succulents. A misreport of DW as FW could also be a possible explanation for that inconsistency.

PLs and GLs are two major lipid groups present in the total lipid extracts of halophyte leaves, carrying a wide array of fatty acids (FAs), from which α-linolenic acid (C18:3, *n*−3), palmitic acid (C16:0), linoleic acid (C18:2, *n*−6) and oleic acid (C18:1, *n-*9) are the most abundant^22,52,53^. In this study, *H. portulacoides* displayed a profusion of lipid species with C16 and C18 chains and some species exhibited polyunsaturated fatty acids with up to four double-bonds (e.g. MGDG 18:2/16:4; PG 16:0/18:4 and PG 18:2/18:4). Polyunsaturated FAs have been largely associated with beneficial health effects in humans and animals^54–58^ and *H. portulacoides* leaves can be a good source for obtaining those FAs, given its relatively high lipid content compared with other halophytes.

The quantities of GLs and PLs in the lipid extract were comparable across treatments. The GLs constituted approximately 46% of the total extract, PLs constituted around 18% and, therefore, polar lipids constituted 64% of the total. When the concentrations were expressed in relation to the dry weight (DW) of leaves, GLs and PLs turned out to be significantly lower in [N,P]_low_ treatment. Higher percentages of polar lipids were previously observed in other halophytes, especially GLs. For instance, the total lipidic extract of chloroplast-enriched portions from *Salicornia perennans* Willd. was reported to have 67% GLs and 31% PLs; *Limonium gmelinii* (Willd.) Kuntze, 60% GLs and 32% PLs; and *Artemisia santonicum* L., 80% GLs and 15% PLs^59^. Other halophytes displayed lower percentages of GLs, such as *Halostachys caspica* C.A.Mey. and *Halocharis hispida* (Schrenk) Bunge, with 22–29% GLs and 16–17% PLs in their extract^60^. In terms of the amounts of GLs and PLs per unit of leaves, a previous study reported values for several halophyte species to range between 5–47 mg GLs g^−1^ DW and 2–17 mg PLs g^−1^ DW^61^. Concerning the present study, *H. portulacoides* from the high-input treatments displayed values very similar to the upper-end of those ranges. Nonetheless, comparisons should be taken merely as an illustration of the range of possible concentrations in the edible portions of different species of halophytes.

Regarding the lipidome, the null hypothesis under test stated that *no changes occur in the polar lipidome of H. portulacoides cultivated in low-, medium- and high-input of DIN and DIP*. Since P is an important element of polar lipids, hydroponic conditions that would offer limited access of this element to the plant could promote alterations in the lipidome of leaves. A decrease in PLs in parallel with an increase in non-phosphorus GLs (e.g. SQDG and DGDG) and betaine lipids (in algae) was observed in plants and algae when exposed to conditions of P-limitation, as previously reported for *Arabidopsis*^62–65^, rice^66^, oat^67^, soybean^66,68^, periphyton^69^ and *Ulva*^70^. From the PCA results it follows that the total lipidome signature of *H. portulacoides* leaves remained relatively unchanged across groups after long-term exposure to nutrient concentrations varying between 6–100 mg DIN-N L^−1^ and 0.8–15.5 mg DIP-P L^−1^. However, a sequential overlap of treatment groups, from lowest to highest P-input ([N,P]_low_ < [N,P]_med_ < [N,P]_high_ < CT), was evident in the PLS-DA projection. The [N,P]_low_ group stood out as the group with the least amount of overlap with the other groups. When GLs were analyzed separately from PLs, both PCA and PLS-DA plots evidently discriminated the [N,P]_low_ group from both the CT and [N,P]_high_ groups. These results suggest that the GL profile of the leaves is changing according to the availability of P, but its effect on the total polar lipidome is masked by the PL profile which remains relatively stable across treatments. Therefore, at low-input concentrations of P, the leaves of *H. portulacoides* display a low degree of lipidome remodulation associated with significant changes in GLs. In plants, GLs are typically found in chloroplast thylakoids, being their major lipid constituents, but under P-limited conditions, GLs (particularly DGDG) can partially replace PLs in extra-plastidial membranes^71^. The upregulation of genes encoding GLs synthase (ex. DGD1, DGD2, MGD2/MGD3) which activate additional GLs biosynthetic pathways in plants under P-limited conditions^71–73^ could explain the changes observed in *H. portulacoides’* GL profile under the conditions of low P-input. Previous studies, in both plants and microalgae, demonstrated that the availability of N also affects the morphology and function of chloroplasts in superior plants and microalgae^74–78^ and an accumulation of GLs can be therefore observed under both N- and P-limitation. The GL species that most contributed to the discrimination of treatment groups in the PLS-DA models (i.e. MGDG 34:3, DGDG 34:3, SQDG 34:3, DGDG 36:3 and SQDG 36:6) also displayed a significantly higher relative abundance in [N,P]_low_ than in either CT or [N,P]_high_.

The PLs that allowed some discrimination between groups were PA 34:1, PA 36:3 PA 34:2, PI 36:6 and PC 36:6. The PAs displayed higher intensity as the input of P increased meanwhile PI 36:6 and PC 36:6 displayed an opposite pattern. This is also evident from PAs relative abundances, as PA 34:1, PA 34:2 and PA 36:3 were more abundant in CT than the other groups, meanwhile C36:6 species were generally in lower abundance in CT. In plants, PAs are precursors of PL and GL synthesis and also function as signal molecules of environmental stress^79^. The marked differences in abundance of several PA species between CT and the other treatments could be related to the activation of different metabolic pathways mediated by the availability of P. Moreover, certain FA configurations were constantly associated with lipid species that displayed significant differences in relative abundance (e.g. C34:1, C34:2, C34:3 and C36:6). For instance, C34:3 displayed the highest abundance in [N,P]_low_ across all classes of PLs (except PA) and GLs. In general, variations in relative abundance were observed most evidently between [N,P]_low_ and both the CT and [N,P]_high_ treatments, suggesting a possible metabolic adaptation from high-input to low-input conditions.

Following the observations discussed above, *H. portulacoides* was probably under some level of nutrient limitation under [N,P]_low_. Firstly, they exhibited less PLs and GLs per unit weight of leaves. Secondly, GLs were suffering some degree of remodulation. Thirdly, the relative abundances of certain species in each class changed as a function of N and/or P availability, as suggested by their gradual increase (or decrease) from low-input to high-input of N and/or P. Nonetheless, one could argue about the extent of nutrient limitation that *H. portulacoides* was potentially exposed to under the [N,P]_low_ treatment, by looking at how other plants behaved in similar conditions. For instance, wild specimens of *Arabidopsis thaliana* (L.) Heynh, exposed to 0.03 mM P (similar to [N,P]_low_) during 12 days followed by 4 days without P, were considered P-starved as they clearly exhibited significant decreases in shoot’s PLs (PC, PE, PG and PS) and significant increases in shoot’s GLs (MGDG, DGDG, SQDG)^80^. Some species of MGDG and DGDG were also found at markedly higher levels in soybean (*Glycine max* (L.) Merr.) leaves under P-limited conditions^68^. In this experiment, *H. portulacoides* did not exhibit such patent changes in the lipidome in the low-input conditions, which indicates that plants were not starved. Note, however, that *H. portulacoides* is a perennial plant and both *A. thaliana* and *G. max* are annual plants, and these different life history strategies might affect nutrient utilization and threshold conditions for nutrient-limitation^81,82^. Another important fact to consider is that the impact of P-limitation might not affect leaves homogeneously. For instance, in *G. max* under P-limitation, there seems to be a mechanism of P-remobilization from older leaves, where differences in the lipidome between limited and non-limited conditions were substantial, to younger leaves, where the lipidome profile between different conditions were very similar^68^. In the present study there was no control regarding leaves’ age, as the lipidome was representative of the total pool of leaves from *H. portulacoides*.

Plants in general display a range of responses to low P, generally referred to as P-starvation responses, that aim to minimize the negative effects of its scarcity in plants (e.g. decreased growth, increased root/shoot ratio, increased root-hair density, increased carboxylate exudation, P-remobilization)^83–87^. Under the conditions of the present experiment, the extent to which *H. portulacoides* underwent a starvation response under [N,P]_low_ that affected the polar lipids of its leaves was defined by a decrease in total GLs and PLs and some degree of lipid remodulation detected in the GLs pool. The availability of P was still high enough in the low-input treatment to maintain the PLs pool relatively unchanged.

Within an IMTA framework, it is fair to conclude that *H. portulacoides* is capable of maintaining a fully stable lipidome across a variety of N and P concentrations typical of aquaculture effluents, specifically 20–100 mg DIN-N L^−1^ and 3–15.5 mg DIP-P L^−1^. At lower concentrations (e.g. [N,P]_low_ values: 6 mg DIN-N L^−1^ and 0.8 mg DIP-P L^−1^) the lipidome of the leaves displays some changes, particularly regarding GLs, as well as generalized decrease in the quantity of polar lipids in the leaves. These changes suggest a metabolic adaptation to the lower nutrient conditions and could be indicative of nutrient limitation. Data on growth performance supports a scenario of nutrient-limited conditions in [N,P]_low_, as *H. portulacoides* exposed to those same concentration of N and P produced less biomass than those exposed to higher concentrations (Custódio *et al*., unpublished data).

*Halimione portulacoides* appears to be a good candidate for IMTA in terms productivity and nutrient-extraction^44,88^ and has the potential to become a valuable co-product with uses in human nutrition^11^ and for other applications^13,22,30^. A note should be made, however, about the possibility of halophytes accumulating undesired compounds if these are present in effluents, like metals^89,90^ and chemicals used for therapy and prophylaxis in aquaculture^91^. This possibility must be taken into account when selecting halophytes for IMTA, since the accumulation of contaminants in edible plant organs can pose risks to human health^92^ and species that do not accumulate or concentrate contaminants mostly in non-edible tissues will be more appropriate from a product-safety perspective. The same concern has been put forward regarding other extractive species (e.g. seaweeds) and changes in regulatory frameworks are necessary to promote safety of new products from IMTA^93,94^.

Determining which nutritional conditions can lead to nutrient-limitation scenarios is important information for future IMTA/halophyte producers, in order to guide nutrition strategies that guarantee a consistent end-product, especially under highly variable nutritional outputs which can occur in aquaculture activities. For researchers, this data can guide the establishment of reference nutritional concentrations for future studies targeting the production of *H. portulacoides*. Future lipidomic studies in *H. portulacoides* should also attempt to characterize and quantify seed oils, since these comprise a significant fraction of the aboveground biomass during the reproductive period of this species and could have valuable high-end applications, such as pharmaceuticals, biofuels, detergents, polymers and cosmetics.

Fully characterizing the diversity of lipid species across *H. portulacoides* tissues and how they change along the production cycle and environmental conditions is of tremendous importance for the commercial exploration of its lipids. This will allow for strategic choices to be made on how to produce it and manipulate its life cycle so to maximize the delivery of value-added compounds with commercial applications and consequently increase its economic value.

## Material and Methods

### Plant material

*Halimione portulacoides* stems were harvested on April 2017 at Ria de Aveiro (mainland Portugal) (40°38′04.1″N 8°39′40.0″W) and 500 grafts with 4 nodes each were cut, put into polyethylene containers, irrigated with a modified Hoagland’s solution and placed under natural sunlight and temperature to promote root development. The elemental composition of the modified Hoagland’s solution was: 60 mg K L^−1^, 56 mg N L^−1^, 40 mg Ca L^−1^, 16 mg Mg L^−1^, 16 mg P L^−1^, 1.12 mg Fe L-1, 0.34 mg Mo L-1, 0.28 mg B L^−1^, 0.13 mg Zn L^−1^, 0.11 mg Mn L^−1^ and 0.03 mg Cu L^−1^. After three months, in July 2017, rooted plants were transferred and acclimated to indoor conditions for two weeks. In the second week, plants were progressively exposed to a target water salinity of 20 ppt, with increments of 5 every second day, prior to the beginning of the experiment.

### Growth trial

The hydroponics growth trial took place indoors at ECOMARE (Laboratory for Innovation and Sustainability of Marine Biological Resources of University of Aveiro) facilities, during 10 weeks (from July to September under an artificial photoperiod of 14 light: 10 dark) to allow plants to develop a harvestable aboveground biomass. The hydroponic units were made of opaque polypropylene material, with dimensions of 300 × 200 × 170 mm and a volume of 5 L of solution maintained through an overflow outlet. Twenty polystyrene floating-rafts were perforated with ten holes equally spaced between them (20 mm) and 200 three-months old rooted grafts of *H. portulacoides* with similar weights were randomly distributed into 20 hydroponic units, at a density of 10 plants per unit. Plants were inserted in the holes by the roots and fixed in place at the lower level of the stem using natural cotton.

The experiment consisted in 4 treatment solutions (including a control) and 5 replicate units (n = 5). The basis for the treatment solutions was an artificial seawater produced by mixing sea salts (Red Sea salt, Red Sea Aquatics, Cheddar, UK) with tap water purified by reverse-osmosis (V2Pure 360 RO System, TMC, Hertfordshirem UK) at a salinity of 20 ppt. At this salinity, the minerals Ca, Mg and K in the base solution are at a concentration of 235–248 mg L^−1^ Ca, 703–742 mg L^−1^ Mg and 213–226 mg L^−1^ K, according to information provided by the manufacturer. The control solution was the modified Hoagland’s solution described above. The low-, medium- and high-input treatments consisted on modified versions of the control, where only nitrogen (N) and phosphorous (P) were adjusted, to mimic aquaculture-like effluents. Nomenclature and concentrations of N and P are as follows: [N,P]_low_ = [6 mg N L^−1^, 0.8 mg P L^−1^]; [N,P]_med_ = [20 mg N L^−1^, 3.0 mg P L^−1^]; [N,P]_high_ = [100 mg N L^−1^, 6.0 mg P L^−1^]; Control = [56 mg N L^−1^, 15.5 mg P L^−1^]. For a detailed molecular and elemental composition of each treatment please see the supplementary Table S1 in the Supplementary Material available online. The solutions within each unit were continuously aerated with a small aerator to keep oxygen levels high and units were refilled with reverse-osmosis water as needed, to compensate for evapotranspiration. The treatment solutions were renewed weekly, as the retention time for nutrient extraction was set to one week. At the end of the growth trial, the leaves of individual plants were cut out, pooled by hydroponic unit and stored at −80 °C until further analysis. Water temperature and pH were measured regularly with a multi-parameter water quality meter and photosynthetically active radiation (PAR) was measured with a spherical micro quantum sensor (US-SQS/L, Heinz Walz GmbH, Pfullingen, Germany). Average values recorded at the end of each week, before renewal of treatment solutions, are presented as supplementary Table S2.

### Analytical methods

#### Reagents

HPLC grade chloroform, methanol and acetonitrile were obtained from Fisher Scientific Ltd. (Loughborough, UK). Lipid internal standards 1,2-dimyristoyl-sn-glycero-3-phosphate (dMPA), 1,2-dimyristoyl-sn-glycero-3-phosphocholine (dMPC), 1,2-dimyristoyl-sn-glycero-3-phosphoethanolamine (dMPE), 1,2-dimyristoyl-sn-glycero-3-phospho-(10-rac-glycerol) (dMPG), 1,2-dipalmitoyl-sn-glycero-3-phosphatidylinositol (dPPI) and 1-nonadecanoyl-2-hydroxy-sn-glycero-3-phosphocholine (LPC) were purchased from Avanti Polar Lipids, Inc. (Alabaster, AL). Milli-Q water (Synergy, Millipore Corporation, Billerica, MA, USA) was produced when ultrapure water was necessary. All other reagents were purchased from major commercial sources.

#### Leaves lipid extraction

Total lipids were extracted according to the method proposed by Bligh & Dyer (1959)^95^, modified for seaweeds and halophytes^22^. 3.75 mL of chloroform: methanol (1:2, v/v) were added to 100 mg of freeze-dried and grounded leaves followed by 2-minutes vortex stirring and 1-minute sonication. Samples were then incubated on an orbital shaker for 2.5 h, on ice. The homogenates were centrifuged at 2000 rpm for 10 -minutes. The chloroform: methanol extraction followed by centrifugation was repeated twice to improve extraction efficiency. After extraction, 2.3 mL of ultrapure water was added to each supernatant, stirred on the vortex and centrifuged at 2000 rpm for 10- minutes. Two liquid phases originate and the inferior organic phase, which contains the lipids, was recovered and dried under a stream of nitrogen gas. Each dried extract was dissolved in 600 µL of chloroform and transferred to dark vials. Lipid extracts were dried under nitrogen gas, weighed (for ‘total lipid’ calculation) and stored at −20 °C prior to LC − MS analysis.

#### Quantification of phospholipids

Quantification of the total phospholipid content was achieved by using the protocol by Bartlett and Lewis (1970)^96^. First, in glass tubes, 125 μl of percloric acid (70 % v/v) was added to dried lipid extracts and the mixtures were incubated during 40 minutes at 170 °C. In the meantime, standards were prepared, also in glass tubes, using 0.1 to 2 μg of phosphorous. After, 825 μl of ultrapure water, 125 μl of ammonium molybdate (2.5 % v/v) and 125 μl of ascorbic acid (10 % v/v) were added to each sample and standards. All tubes were then vortexed. Tubes were incubated in water bath at 100 °C for 10 minutes and transferred to ice to cool down. Absorbance of samples and standards were measured at 797 nm using a microplate reader (Multiskan GO, Thermo Scientific, Hudson, NH, USA).

#### Quantification of glycolipids

Quantification of the total glycolipid content was achieved using the orcinol assay, as done in our lab^25,97^. First, an orcinol solution (0.2 % v/v in 70 % sulfuric acid) was prepared and 1 mL was added to tubes with N_2_-dried lipid extract samples. Tubes were heated at 80 °C for 20 minutes and transferred to ice to cool down. Absorbance of samples and standards were measured at 505 nm using a microplate reader (Multiskan GO, Thermo Scientific, Hudson, NH, USA). The concentration of glucose was calculated by comparing the data with those of glucose standards (between 0–50 μg prepared from an aqueous solution containing 2 mg mL^−1^ of glucose and following the same procedure as experimental samples).

#### Analysis of polar lipids by high resolution LC-MS and MS/MS

The polar lipids from *H. portulacoides* leaves were analyzed by high−performance LC (HPLC) system (Thermo Scientific Accela, Thermo Fisher Scientific, USA) with an autosampler coupled online to the Q-Exactive® mass spectrometer with Orbitrap® technology following the method previously used for halophyte lipid analysis^22^. The solvent system consisted of two mobile phases: mobile phase A [acetonitrile:methanol:water 50:25:25 (v/v/v) with 1 mM ammonium acetate] and mobile phase B [acetonitrile:methanol 60:40 (v/v) with 1 mM ammonium acetate]. Initially, 0% of mobile phase A was held isocratically for 8 minutes, followed by a linear increase to 60% of A within 7 minutes and a maintenance period of 15 minutes, returning to the initial conditions within 10 minutes. A volume of 5 µL of each sample containing 20 µg of lipid extract, a volume of 4 µL of internal standards mix (dMPA − 0.02 µg µg^−1^; dMPC − 0.005 µg µg^−1^, dMPE − 0.005 µg µg^−1^, dMPG − 0.003 µg µg^−1^, dPPI − 0.02 µg µg^−1^, LPC − 0.005 µg µg^−1^) and 91 μL of mobile phase B were pipetted and introduced into the Ascentis^®^Si column (15 cm × 1 mm, 3 μm, Sigma-Aldrich) with a flow rate of 40 μL min^−1^ at 30 °C. The mass spectrometer with Orbitrap® technology was operated in simultaneous positive (electrospray voltage 3.0 kV) and negative (electrospray voltage −2.7 kV) modes at a resolution of 70,000 and AGC target of 1e6, the capillary temperature was 250 °C and the sheath gas flow was 15 U. In MS/MS experiments, a resolution of 17,500 and AGC target of 1e5 was used and the cycles consisted of one full scan mass spectrum and ten data-dependent MS/ MS scans, repeated continuously throughout the experiments with a dynamic exclusion of 60 seconds and intensity threshold of 1e4. Normalized collision energy™ (CE) ranged between 25, 30 and 35 eV. MZmine 2.27 software was used to process MS raw data and identify lipid species by mass accuracy from high resolution MS data. Thermo Xcalibur 3.0.63 software was used to analyze the chromatograms and MS/MS spectra, to confirm lipid species identity and discriminate their fatty-acid composition. The classes lysophosphatidylethanolamine (LPE), phosphatidic acid (PA), phosphatidylethanolamine (PE), phosphatidylglycerol (PG), phosphatidylinositol (PI) and sulfoquinovosyldiacylglycerol (SQDG) were detected as anionized adducts of [M-H]^−^; digalactosyldiacylglycerol (DGDG), monogalactosyldiacylglycerol (MGDG) and monogalactosylmonoacylglycerol (MGMG) were detected as cationized adducts of [N + NH_4_]^+^; and lysophosphatidylcholine (LPC) and phosphatidylcholine (PC) were detected as cationized adducts of [M + H]^+^. The FA composition of PCs and LPCs were identified by analysis of the MS/MS of anionized adducts of acetate [M + Ac]^−^, which detects the carboxylate anions R-COO^−^ that allow the determination of the fatty acyl composition. The dataset with the peak intensities, normalized to internal standards, is available online in the spreadsheet “Supplementary Dataset 1”.

### Statistical analysis

Statistical analysis was performed using R (v3.4.3) in combination with RStudio (v1.1.463) and MetaboAnalyst (v4.0)^98^. Prior to analysis, the lipidomic dataset was normalized by dividing peak-intensity values of each molecular species with the peak-intensity of their respective internal standard (Supplementary Dataset 1). Secondly datasets were created for each lipid class, where the relative abundance of each molecular species was computed for each replicate (Supplementary Dataset 2).

Prior to the multivariate analysis, data normalization procedures - *log-transformation* followed by *auto-scaling* - were employed to decrease the influence of high-concentration metabolites and increase the statistical strength of low-concentration metabolites. Both unsupervised (Principal Components Analysis - PCA) and supervised (Partial Least Squares Discriminant Analysis - PLS-DA) methods were used.

The univariate analysis consisted on the analysis of variance, using the non-parametric Kruskal-Wallis test, of species i) peak-intensities and ii) relative abundance within each class. *Post-hoc* Dunn’s test was used for pairwise comparisons and the Benjamini-Hochberg method was used to control for type-I errors^99^. Significant differences were assumed at a critical p-value <0,05.

## Supplementary information

Supplementary material.

Supplementary Dataset 1.

Supplementary Dataset 2.

## Data Availability

All data generated or analyzed during this study are included in this published article and its Supplementary Material files.
