# Supplementary material for: Nutrient availability affects the polar lipidome of Halimione portulacoides leaves cultured in hydroponics
Source: Sci Rep. 2020 Apr 20;10:6583. doi: 10.1038/s41598-020-63551-1 (PMC7171145; doi:10.1038/s41598-020-63551-1)

Supplementary Material

for

Nutrient availability affects the polar lipidome of *Halimione portulacoides* leaves cultured in hydroponics

Marco Custódio*, Elisabete Maciel, M. Rosário Domingues, Ana I. Lillebø; Ricardo Calado*

*Corresponding authors:

E-mail: mfc@ua.pt; Tel.: +351913105645 (M Custódio)
E-mail: rjcalado@.pt; Tel.: +351234370779 (R Calado)

Table S1: Molecular (I) and elemental (II) composition of treatment solutions. Note: values do not account for the additional elements in the artificial saline water used as base-solution.

**Molecular composition**	**Molar mass** (g/mol)	**$[N,P]_{low}$** (mM)	**$[N,P]_{med}$** (mM)	**$[N,P]_{high}$** (mM)	**Control** (mM)
**KNO_3**	101.11	0.40	1.32	1.50	1.50
**$Ca(NO_3)_2.4H_2O$**	236.15	-	-	-	1.00
**$NH_4H_2PO_4$**	115.03	0.03	0.10	0.20	0.50
**$MgSO_4 . 7H_2O$**	246.48	0.25	0.25	0.25	0.25
**KCl**	74.56	1.155	0.23	0.05	0.05
**H_3BO_3**	61.83	0.03	0.03	0.03	0.03
**$MnSO_4 . H_2O$**	169.01	2e-3	2e-3	2e-3	2e-3
**$ZnSO_4 .7H_2O$**	287.56	2e-3	2e-3	2e-3	2e-3
**$CuSO_4 . 5H_2O$**	249.68	5e-4	5e-4	5e-4	5e-4
**$(NH_4)_6Mo_7O_{24} . 4H_2O$**	1235.86	5e-4	5e-4	5e-4	5e-4
**FeNaEDTA**	367.05	0.02	0.02	0.02	0.02
**NH_4NO_3**	80.04	-	-	2.65	-

**Elemental composition**	**Atomic mass**	**[N,P]low** (ppm)	**[N,P]med** (ppm)	**[N,P]high** (ppm)	**Control** (ppm)
**N**	14.00	5.92	19.92	98.04	56.04
**P**	30.97	0.77	3.10	6.19	15.49
**S***	32.06	8.16	8.16	8.16	8.16
**K***	39.10	60.61	60.61	60.61	60.61
**Ca***	40.08	-	-	-	40.08
**Mg***	24.31	6.08	6.08	6.08	6.08
**Fe**	55.85	1.12	1.12	1.12	1.12
**Cu**	63.55	0.03	0.03	0.03	0.03
**Zn**	65.38	0.13	0.13	0.13	0.13
**Mn**	54.94	0.11	0.11	0.11	0.11
**B***	10.81	0.27	0.27	0.27	0.27
**Mo**	95.95	0.34	0.34	0.34	0.34

***** These elements are present in very high concentrations in the artificial saline water. Elemental variations between treatments (which only occurred in Ca) due to N and P adjustments are therefore considered negligible. At 20 ppt, Red Sea® saline water has, according to manufacturer's information: 235-248 mg L^{-1} Ca; 703-742 mg L^{-1} Mg and 213-226 mg L^{-1} K.

Table S2: Average water temperature, pH and photosynthetically active radiation (PAR) at the end of each remediation week.

	Week									
	**1**	**2**	**3**	**4**	**5**	**6 †**	**7**	**8**	**9**	**10**
**Temperature** (ºC)	23.5 ± 0.1	23.1 ± 0.0	21.5 ± 0.1	22.5 ± 0.1	22.6 ± 0.0	25.0 ± 0.2	20.9 ± 0.3	21.3 ± 0.0	21.2 ± 0.0	21.5 ± 0.1
**pH**	7.7 ± 0.0	7.7 ± 0.0	7.7 ± 0.0	7.5 ± 0.0	7.7 ± 0.1	6.7 ± 0.1	7.1 ± 0.7	7.0 ± 0.6	6.9 ± 0.7	7.1 ± 0.6
**PAR** (µmol s^{-1} m^{-2})	332.0 ± 54.8	303.5 ± 44.8	321.3 ± 55.1	315.0 ± 42.0	331.3 ± 34.0	333.8 ± 47.5	356.3 ± 53.4	350.0 ± 66.3	303.8 ± 53.4	301.3 ± 61.4

† A drop in pH to values between 6.6 - 6.8 was registered in all treatments at the end of week 6. This occurrence coincided with an abrupt increase in air temperature in the facilities due to a failure in the ventilation system. Plants were exposed to this increase in room temperature for a maximum of 16 hours overnight. Treatment solutions were renewed the next day, coinciding with the end of a nutrient extraction period.

Figure S1: PCA loadings plot of A) total lipidome, B) phospholipids and C) glycolipids

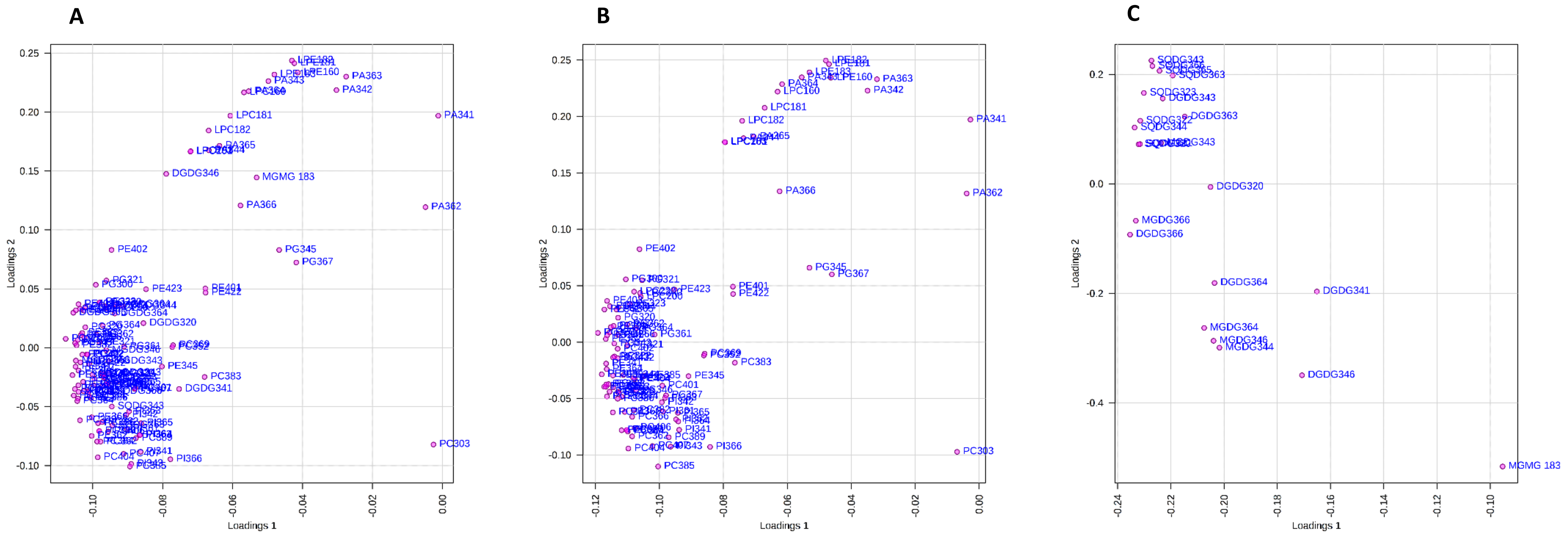

Figure S2: Univariate non-parametric Kruskal Wallis plot of peak-intensities (red dots - $p < 0.05$) and box-whiskers plots of significantly different species (* - $p < 0.05$).

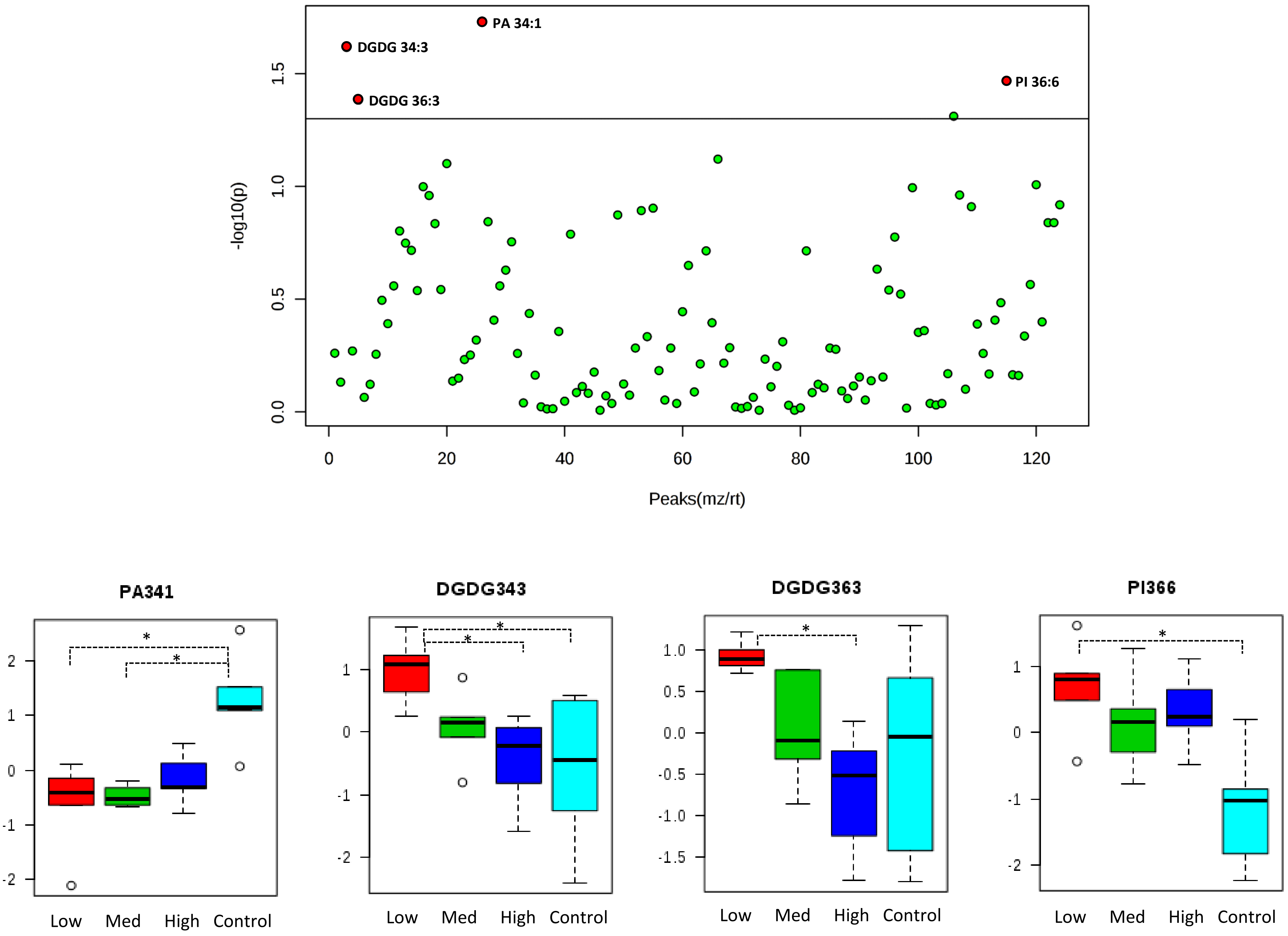

Figure S3: MS/MS spectra from all conditions of top VIP (Variable Importance in Projection) features in the 'total lipidome' PLS-DA projection: A) PA 34:1, B) PI 36:6 and C) DGDG 34:3, D) MGDG 34:3, E) PC 36:6.

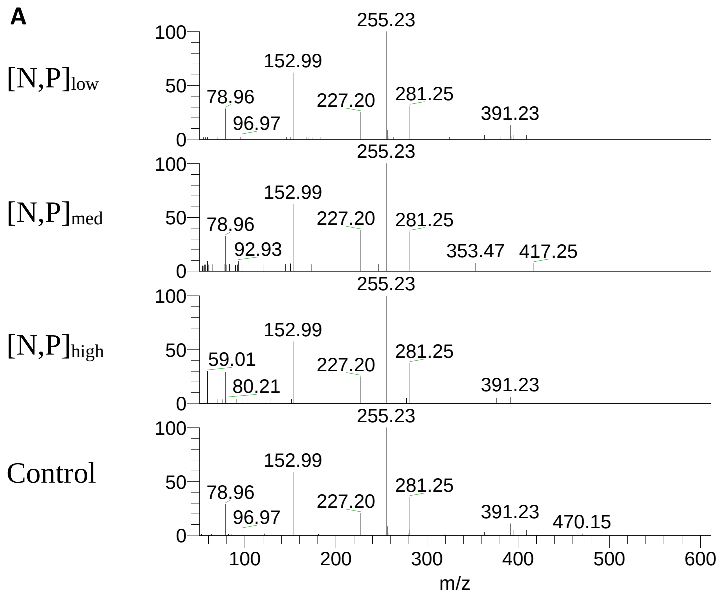

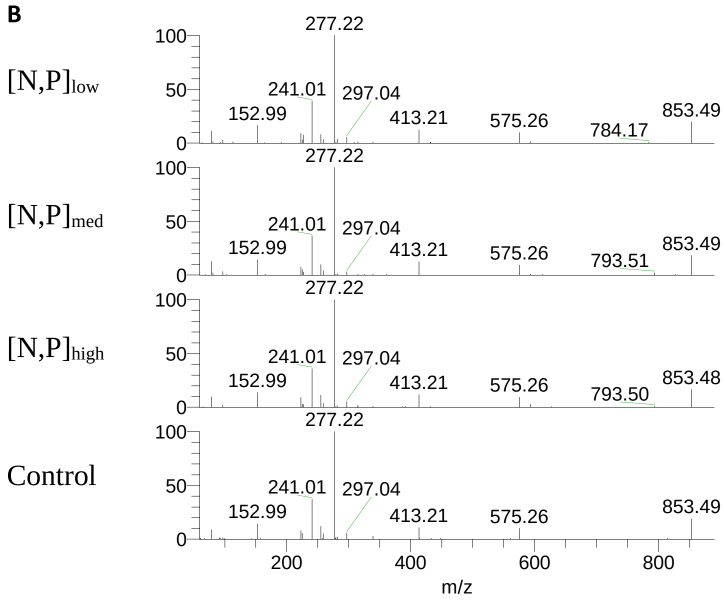

C

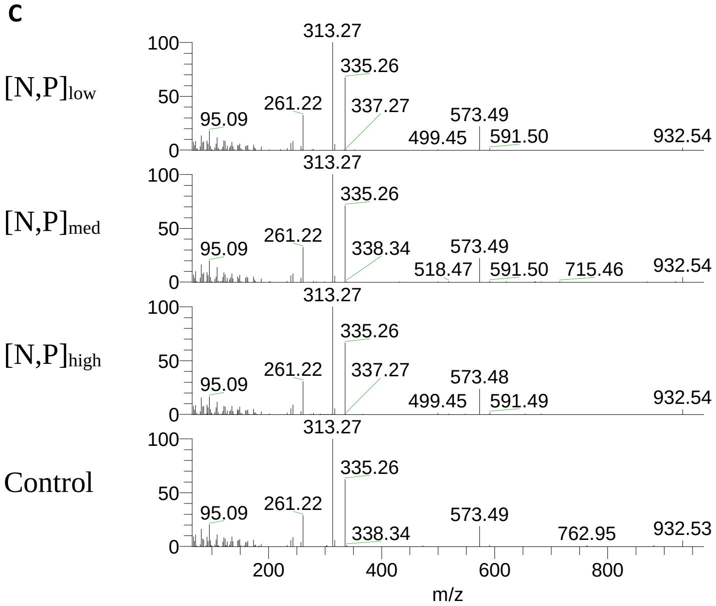
[N,P]low
[N,P]med
[N,P]high
Control
313.27
335.26
337.27
338.34
261.22
95.09
573.49
573.48
499.45
591.50
591.49
518.47
715.46
762.95
932.54
932.53
100
50
0
200
400
600
800
m/z

D

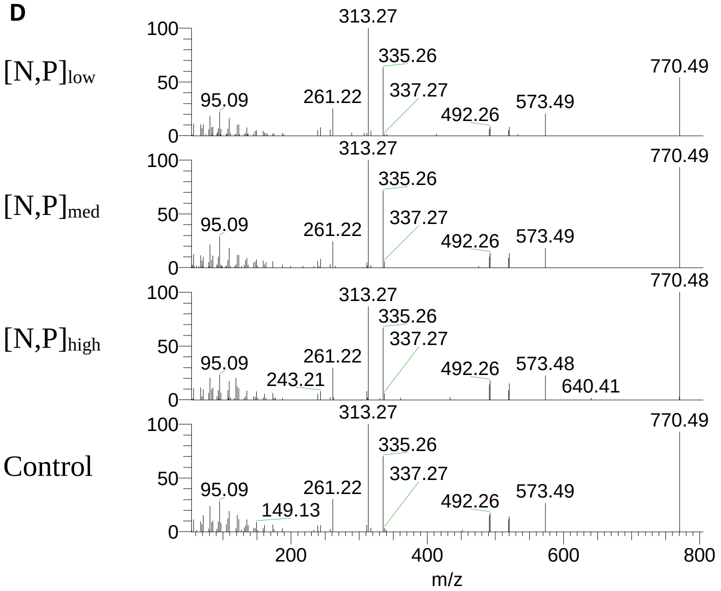
[N,P]low
[N,P]med
[N,P]high
Control
313.27
335.26
337.27
261.22
243.21
149.13
95.09
492.26
573.49
573.48
640.41
770.49
770.48
100
50
0
200
400
600
800
m/z

E

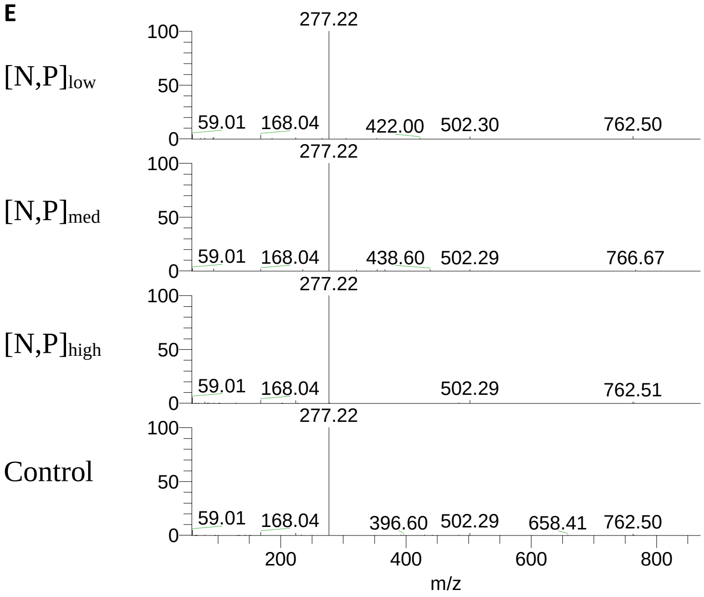
[N,P]low
[N,P]med
[N,P]high
Control
277.22
59.01
168.04
422.00
438.60
396.60
502.30
502.29
658.41
762.50
766.67
762.51
100
50
0
200
400
600
800
m/z

Figure S4: Relative abundance of phospholipid molecular species identified after LC-MS analysis. The results are expressed as a percentage, obtained by dividing the normalized peak areas of each molecular species by the sum of the total peak areas. Error bars represent standard deviations and horizontal lines represent significant differences: * p < 0,05; ** p < 0,01.

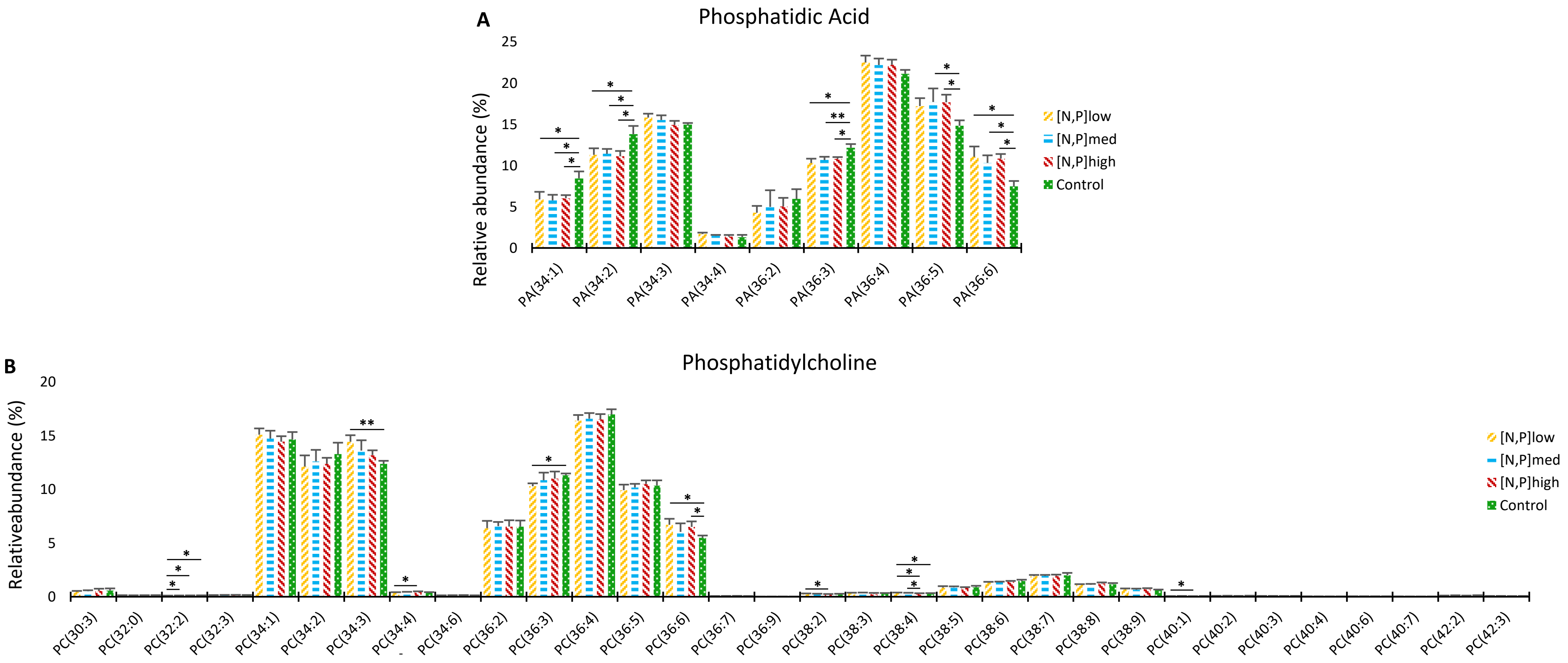

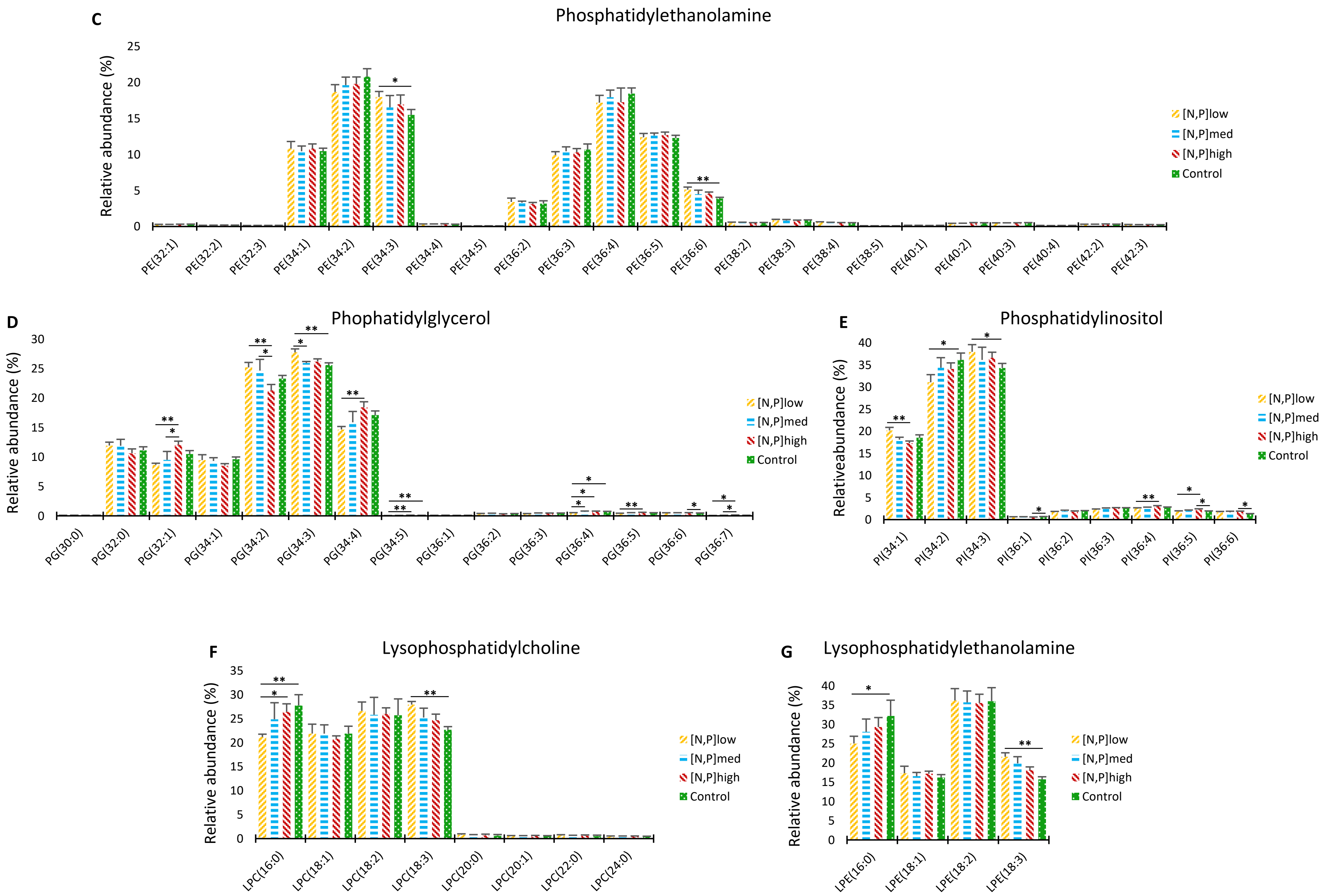

C
Phosphatidylethanolamine
Relative abundance (%)
PE(32:1) PE(32:2) PE(32:3) PE(34:1) PE(34:2) PE(34:3) PE(34:4) PE(34:5) PE(36:2) PE(36:3) PE(36:4) PE(36:5) PE(36:6) PE(38:2) PE(38:3) PE(38:4) PE(38:5) PE(40:1) PE(40:2) PE(40:3) PE(40:4) PE(42:2) PE(42:3)
[N,P]low
[N,P]med
[N,P]high
Control
D
Phophatidylglycerol
Relative abundance (%)
PG(30:0) PG(32:0) PG(32:1) PG(34:1) PG(34:2) PG(34:3) PG(34:4) PG(34:5) PG(36:1) PG(36:2) PG(36:3) PG(36:4) PG(36:5) PG(36:6) PG(36:7)
E
Phosphatidylinositol
Relativeabundance (%)
PI(34:1) PI(34:2) PI(34:3) PI(36:1) PI(36:2) PI(36:3) PI(36:4) PI(36:5) PI(36:6)
F
Lysophosphatidylcholine
Relative abundance (%)
LPC(16:0) LPC(18:1) LPC(18:2) LPC(18:3) LPC(20:0) LPC(20:1) LPC(22:0) LPC(24:0)
G
Lysophosphatidylethanolamine
Relative abundance (%)
LPE(16:0) LPE(18:1) LPE(18:2) LPE(18:3)

Figure S5: Relative abundance of glycolipid molecular species identified after LC-MS analysis. The results are expressed as a percentage, obtained by dividing the normalized peak areas of each molecular species by the sum of the total peak areas. Error bars represent standard deviations and horizontal lines represent significant differences: * p < 0,05; ** p < 0,01.

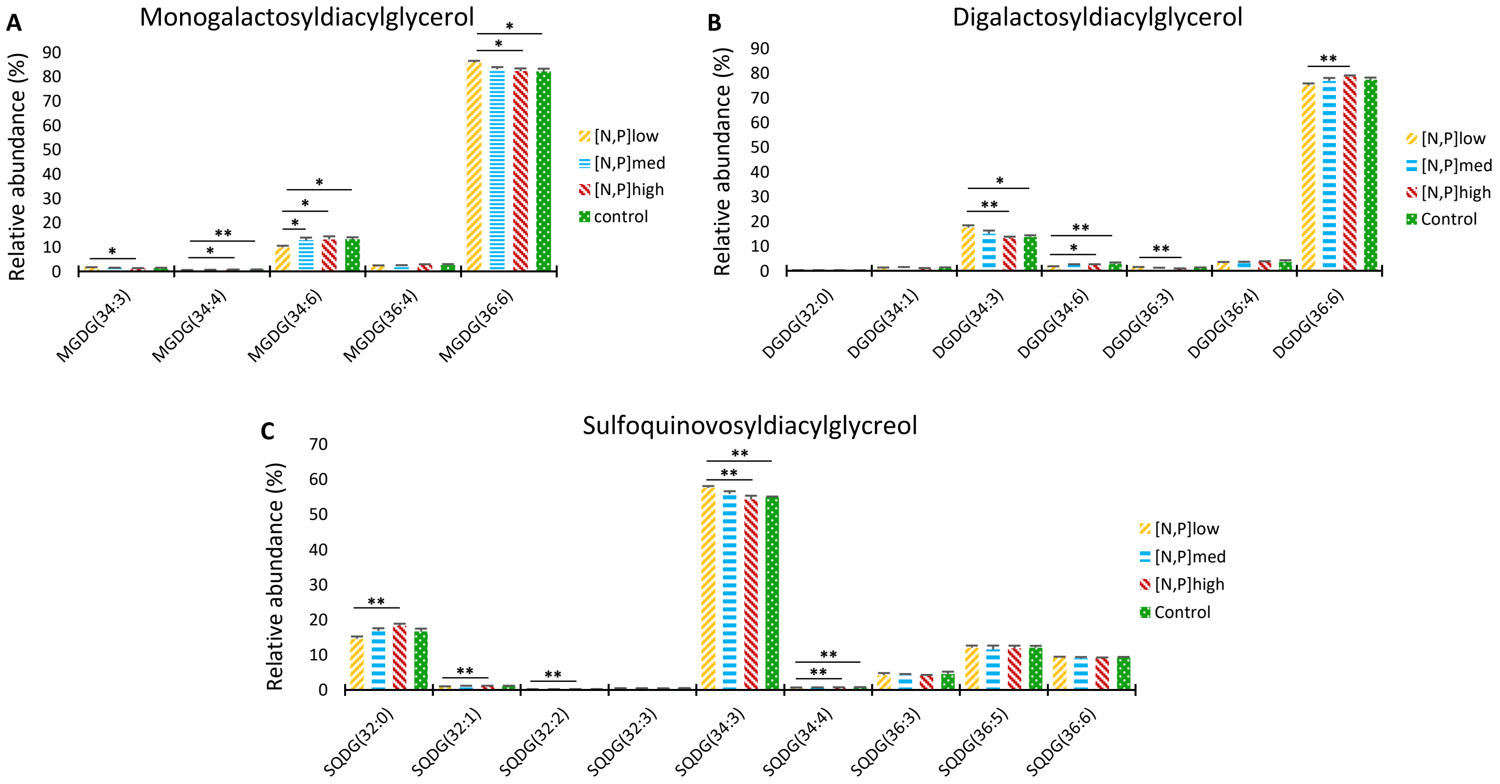

Supplement: Supplementary file 1 — Supplementary material. [file 41598_2020_63551_MOESM1_ESM.pdf]
